# Evaluation of adult dTPaP vaccination coverage in France: experience in Lyon city, 2010–2011

**DOI:** 10.1186/1471-2458-12-940

**Published:** 2012-11-01

**Authors:** Dominique Baratin, Corinne Del Signore, Jacques Thierry, Evelyne Caulin, Philippe Vanhems

**Affiliations:** 1 Hospices Civils de Lyon, Hôpital Edouard Herriot, Service d’Hygiène, Epidémiologie et Prévention, Lyon, France; 2 Institut d’Epidémiologie, Laboratoire de Biométrie et Biologie Evolutive, UMR CNRS 5558, Claude Bernard Lyon 1 University, Lyon, France; 3 Groupement de laboratoires de biologie médicale libéraux DYOMEDEA, Lyon, France; 4 Sanofi Pasteur MSD, Lyon, France

**Keywords:** Vaccination coverage, Adults, Diphtheria, Tetanus, Poliomyelitis, Pertussis

## Abstract

**Background:**

Compliance with official recommendations can be assessed by evaluating vaccination coverage (VC) in populations. The main objective of our study was to assess VC of adults against diphtheria, tetanus, poliomyelitis and pertussis (dTPaP) according to age. The second objective was to explore if vaccination status could be confirmed by documentation.

**Methods:**

A cross-sectional study was conducted in 680 adults consulting for biological examination in private laboratories in Lyon (France) to evaluate VC for diphtheria, tetanus, poliomyelitis and pertussis (dTPaP) and enabled reported vaccinations to be compared with documented, confirmed vaccinations.

**Results:**

Verification of documented, confirmed vaccinations disclosed VC of 78.7% for tetanus, 63.6% for poliomyelitis, 57.8% for diphtheria and 10.7% for pertussis. Comparison of confirmed and self-reported vaccinations revealed that a large percentage of people who thought that they were vaccinated were not. VC significantly decreased with age for diphtheria and poliomyelitis and did not vary by gender. The VC rate for pertussis has increased since the 2008 recommendations were made.

**Conclusions:**

The main thrust of this study was to compare reported and confirmed data. A significant percentage of people wrongly believed that they were up to date with their vaccination.

## Background

Vaccination recommendations are updated in line with changes in epidemiological data, the efficacy and tolerability of existing vaccines, and the advent of new vaccines on the market. Studies in France and Europe [1,2] have highlighted the need to improve vaccination policies. Ignorance of the exact level of vaccination coverage (VC) in the general population does not facilitate comparisons over time or evaluation of vaccination impact. VC monitoring of at-risk populations and within specific age groups would be useful, aided by the establishment of indicators in the general population for the main vaccination-preventable diseases. Available sources of information are personal child health records for young adults, new personal adult health records, university files, international vaccination records, occupational health records, medical records, medical insurance companies and prescribing physicians.

A sero-epidemiological investigation of adults reported that the percentage of subjects with diphtheria and tetanus antibodies decreased with age and that 7.6% of the population studied (13.4% of those aged 18–29 years) had recently been infected by pertussis [3]. These results underline the need for better application of the recommendations for pertussis booster vaccines in adults [4]. The lack of any systematic follow-up of VC and the heterogeneous nature of various practices [5] might partly explain such observations. The population’s perception of vaccination is also a determining factor, and individuals’ knowledge of their vaccination status is probably not optimal.

The primary objective of our study was to evaluate adult diphtheria, tetanus, polio and pertussis (dTPaP) VC by age in the general population. The secondary objectives were to compare self-reported dTPaP VC with documented VC obtained from vaccination records and to verify dTPaP vaccination in adults.

## Methods

### Study setting and data collection

Our cross-sectional study was conducted in co-operation with 6 private medical analysis laboratories located in different districts of Lyon, a city with more than 480,000 inhabitants. Each laboratory covered about 14,000 inhabitants. Laboratory participation was voluntary and previously effective in collaborative academic epidemiological studies [5].

Subjects included in the survey were aged over 19 years and had to have attended a medical analysis laboratory for tests of any kind (glycaemia, serology, etc.). All patients were contacted but only those volunteering to participate were enrolled. After informing each study subject and obtaining signed consent, research assistants collected vaccination data with a standardised questionnaire complemented by socio-demographic data. Vaccination documentation was always requested. Data on subjects’ knowledge of their vaccination status focused on diphtheria, tetanus, poliomyelitis, pertussis, and the conditions under which these vaccinations were given. Questions were asked in relation to pertussis: intention to become parents, pregnancy, contact with children under 6 months old and knowledge of the disease. Vaccination data were validated by documentary “evidence” of vaccination. Descriptive analysis was performed relative to socio-demographic factors and vaccination data supported by documentary “evidence”. Subjects vaccinated within the past 10 years were considered to be up to date with their vaccination according to the 2011 French adult immunization schedule (Table 1).

**Table 1:** 2011 French immunisation schedule – Recommendations for adults

							
General recommendations	Vaccines	18-23 years	24 years	26-28 years	30-45 years	46-64 years	≥65 years
	Diphtheria (d) Tetanus (T) Poliomyelitis (IPV)			dT-IPV to be replaced by a single dose of dTaP-IPV for adults who have not received pertussis vaccination since 10 years	1 dose of dT-IPV every 10 years		
	Acellular pertussis (aP)						
Catch-up program	Acellular pertussis (aP)				Replaced by a single dose of dTaP-IPV for adults who have not previously received pertussis vaccination		
Specific and at-risk populations	Acellular pertussis (aP)	A single dose of dTaP-IPV in adults with the prospect of becoming parents (cocoon), family members during pregnancy and post-partum women (minimum interval of 2 years between 1 dose of dT-IPV and dTaP-IPV					

Our survey was conducted from October 13, 2010 to February 11, 2011, after approval by the Advisory Committee on Data Processing in Medical Research and the French National Data Protection Committee.

### Statistical analysis

Qualitative variables were reported as number and percentage, and quantitative variables, as means and standard deviation. VC was calculated for 100 subjects with 95% confidence interval (95% CI). Categorical variables were compared by the Chi square test or Fisher’s exact test as appropriate (less than 5 individuals by cell in 2X2 tables). All data were analysed anonymously with SPSS 17.0 software for Windows (SPSS Statistics Inc., Chicago, IL, USA).

## Results

### Study population characteristics

A total of 680 subjects, aged over 19 years, were surveyed among all patients who attended 6 laboratories during the study period. Median age was 60 years (min: 20, max: 91), and the gender ratio (M/F) was 0.68. Pensioners (56.9%) were the most commonly-represented occupational group, followed by business executive and managers (11.6%), the unemployed, disabled or students (8.0%), healthcare professionals (9.3%), labourers (3.1%), artisans-shopkeepers-business people (0.9%) and farmers (0.4%). Master’s degree (20.6%) was the most frequently-found educational level. Healthcare professionals who administered vaccinations were, in the great majority of cases, family physicians (77%), vaccination centres (9.6%), occupational physicians (8.8%), hospital centres and nurses (0.8%).

In the specific case of pertussis, 15.6% of subjects intended to become parents shortly after the survey, 8.2% were pregnant (or their partner was), and 19.1% had frequent contact with children under 6 months of age. On interview, a third of people who intended to become parents, a third of pregnant women (or their partners) and a third of people in contact with children under 6 months of age thought about getting vaccinated against pertussis. Twenty-nine percent were aware of the value of pertussis vaccination, and 21% of them knew how serious pertussis infection was in adults.

Finally, a total of 33.1% (225/680) of those surveyed reported having vaccination documentation. This documentary evidence assumed different forms: vaccination record (52.9%), vaccination card or certificate (34.2%), and personal health record (12.9%). Table 2 describes the characteristics of study subjects and the Lyon city population.

**Table 2:** Characteristics of individuals with documentation and of the Lyon city population for dTPaP VC evaluation, October 2010-February 2011

**Characteristics**	**Study population n=225**	Lyon city population * ≥20 years n=372,583
**Age (years)**		
Median [IQR**]	63 [51–73]	40 [28–58]
Strata		
20-40	15.1 %	47%
41-60	27.1%	28%
61-80	46.7 %	17%
>80	11.1 %	6%
**Sex**		
Male	35.6 %	46%
Female	64.4 %	54%
**Socio-professional categories**		
Pensioners	59.9%	19.2%
Employees	9.8%	15.4%
Business executive or managers	11.6%	16.9%
Unemployed, student or disabled	8.0%	21.1%
Healthcare professionals	9.3%	17.4%
Labourers	3.7%	7.6%
Artisans, shopkeepers, business people	0.9%	2.5%
Farmers	0.4%	0.0%
**Educational level**		
Master’s degree level 2 (Bachelor + ≥3)	30.2%	28.4%
Master’s degree level 1 (Bachelor + 2)	12.4%	15.9%
Bachelor	20.9%	15.9%
GCSE***, Certificate in vocational training, Technical School Certificate	20.9%	18.9%
Basic school-leaving qualification	12.0%	6.8%
No schooling	3.6%	13.9%
**Number of children**		
0	20.9%	48.2%
1	16.4%	24.1%
2	36.4%	18.7%
3	16.1%	6.6%
≥4	10.1%	2.4%

* data from the french national agency for demographic data ( http://www.insee.fr).

** InterQuartile range *** General Certificate of Secondary Education.

Concerning self-reported vaccination, those who produced documentation, as opposed to those who did not, were predominantly women (64.4% *vs* 56.7%, p=0.05), older people (mean age: 60.7 vs 55.5 years, p=0.001) and people who thought they were up to date with their vaccinations apart from pertussis vaccination (Table 3).

**Table 3:** Self-reported vaccination of Lyon city subjects producing documentation or not, October 2010-February 2011

**Self-reported vaccinations**	**Subjects not producing documentation**	**Subjects producing documentation**	**p**	**Total subjects included**
Sex n (%)				
Female	258 (56.7)	145 (64.4)	<10^-3^	403 (59.3)
Male	197 (43.3)	80 (35.6)		277 (40.7)
Age (years)				
Mean (±SD)	55.5 (± 19.1)	60.7 (± 16.1)	0.001	57.2 [± 18.3]
Median [min-max]	57 [20–91]	63 [21–87]	-	60 [20–91]
Age strata				
20-40	123 (27.0)	34 (15.1)	0.02	157 (23.1)
41-60	129 (28.4)	61 (27.1)		190 (27.9)
61-80	159 (34.9)	105 (46.7)		264 (38.8)
>80	44 (9.7)	25 (11.1)		69 (10.1)
Subjects reporting being up to date with their vaccinations n (%) [95% CI of %]	274 (60.2) [55.9-68.7]	183 (81.3) [75.8-86.0]	<10^-3^	457 (67.2) [63.3-70.7]
Subjects reporting being up to date with each vaccination n (%) [95% CI of %]				
Diphtheria	293 (64.4) [59.9-68.7]	158 (70.2) [64.0-75.9]	0.002	51 (66.3) [62.7-69.8]
Tetanus	383 (84.4) [80.6-87.3]	215 (95.6) [92.9-97.7]	<10^-3^	599 (88.1) [85.5-90.4]
Poliomyelitis	306 (67.3) [62.8-71.4]	171 (76.0) [61.1-72.6]	0.001	477 (70.1) [66.6-73.5]
Pertussis	105 (23.1) [19.4-27.1]	50 (22.2) [15.1-24.8]	0.96	155 (22.8) [19.8- 26.1]

### Vaccine coverage by age

Verification of vaccinations confirmed by documentary evidence (n=225) showed VC of 57.8% for diphtheria, 78.7% for tetanus, 63.6% for poliomyelitis and 10.7% for pertussis (Table 4). VC for each of the 4 valences studied did not vary with gender. VC for diphtheria and poliomyelitis, but not for tetanus or pertussis, decreased significantly with age (Table 4). For confirmed vaccinations, 128 people received combined dT-IPV vaccination (vaccines injected on the same day and in a single dose: dT-IPV and dTaP-IPV are the most commonlyadministered vaccines in France). VC with combined vaccination did not differ significantly with gender, but decreased with age. Some people received a single valence (tetanus), for which a VC gradient with age was observed (Table 4).

**Table 4:** Vaccination coverage and vaccine combination by age, Lyon city, October 2010-February 2011

**Vaccination given within 10 years**	**n=225**	**Non-vaccinated subjects n (Yes %) [95% CI of %]**	**Vaccinated subjects n (Yes %) [95% CI of %]**	p*
Diphtheria		92 (42.2) [35.9-48.7]	130 (57.8) [51.2-64.1]	0.001
20-40 years	34	9 (26.5) [13.8-43.1]	25 (73.5) [56.9-86.3]	
41-60 years	61	21 (34.4) [23.3-46.9]	40 (65.6) [53.0-76.7]	
61-80 years	105	49 (46.7) [37.3-56.2]	56 (53.3) [43.7-62.7]	
>80 years	25	16 (64.0) [44.1-80.8]	9 (36.0) [19.2-57.5]	
Tetanus		48 (21.3) [16.4-27.1]	177 (78.7) [72.9-83.9]	0.83
20-40 years	34	9 (26.5) [13.8-43.1]	25 (73.5) [56.9-86.3]	
41-60 years	61	14 (23.0) [13.7-34.7]	47 (77.0) [65.3-86.3]	
61-80 years	105	14 (13.3) [7.8-20.8]	91 (86.7) [79.1-92.2]	
>80 years	25	11 (44.0) [25.7-63.6]	14 (56.0) [36.4-74.3]	
Polio		82 (36.4) [30.4-42.9]	143 (63.6) [57.1-69.7]	0.005
20-40 years	34	9 (26.5) [12.9-44.4]	25 (73.5) [55.6-87.1]	
41-60 years	61	18 (29.5) [18.5-42.6]	43 (70.5) [57.4-81.5]	
61-80 years	105	39 (37.1) [27.9-47.1]	66 (62.9) [52.9-72.1]	
>80 years	25	16 (64.0) [42.5-83.0]	9 (36.0) [17.9-57.5]	
Pertussis		201 (89.3) [84.8-92.9]	24 (10.7) [7.1-15.2]	0.34
20-40 years	34	30 (88.2) [72.6-96.7]	4 (11.8) [3.3-27.5]	
41-60 years	61	54 (88.5) [77.8-95.3]	7 (11.5) [4.7-22.2]	
61-80 years	105	92 (87.6) [79.7-93.3]	13 (12.4) [6.7-20.3]	
>80 years	25	25 (100)	0 (0)	
dT-IPV **		97 (43.1) [36.8-49.7]	128 (56.91) [50.4-63.3]	0.01
20-40 years	34	9 (26.5) [13.8-43.1]	25 (73.5) [56.9-86.3]	
41-60 years	61	21 (34.4) [23.3-46.9]	40 (65.6) [53.0-76.7]	
61-80 years	105	51 (48.6) [39.1-58.1]	54 (51.4) [41.9-60.9]	
>80 years	25	16 (64.0) [44.1-80.8]	9 (36) [19.2-57.5]	
Tetanus alone		187 (83.2) [77.8-87.8]	38 (16.8) [12.4-22.2 ]	0.01
20-40 years	34	34 (100.0)	0 (0)	
41-60 years	61	56 (91.8) [82.8-96.3]	5 (8.2) [2.7-18.1]	
61-80 years	105	77 (73.3) [64.3-81.1]	28 (26.7) [18.9-35.7]	
>80 years	25	20 (80.0) [61.1-92.3]	5 (20) [6.8-40.7]	

*Statistical difference between those who were vaccinated and those who were not.

**Diphtheria, tetanus and poliomyelitis combined.

Since the publication of pertussis recommendations in March 2008, VC rates appear to have increased (data not reported) but no pregnant women (0/11) in our study were up to date with their pertussis vaccination. No significant difference for pertussis vaccination appeared between men and women (p: 0.50). Moreover, 4% (1/26) of subjects intending to become parents were vaccinated, and 16% (6/36) of those in contact with infants under 6 months of age were vaccinated.

### Vaccine status according to documentation reported or not

Subjects who thought the most that they were up to date with vaccination, compared to those were not, provided documentation (81%/60%, p<0.001). In total, people with documentation believed that they were up to date with their vaccinations, 70.2% for diphtheria, 95.6% for tetanus, 76.0% for poliomyelitis, and 22.2% for pertussis.

Comparison of confirmed and reported vaccinations by subject revealed that some who thought they were vaccinated for diphtheria; tetanus; poliomyelitis and pertussis were actually not (Table 5). Conversely, some subjects who thought they were not vaccinated were up to date with their vaccinations.

**Table 5:** Reported vaccinations (RV) versus confirmed vaccinations (CV), Lyon city, October 2010-February 2011

**Subjects reporting documentation n=225**	**RV n**	**CV n**	RV-CV/CV*Yes% [95% CI of %]
Diphtheria	158	100	36.7% [29.5-44.4]
Tetanus	215	171	20.5% [15.5-26.3]
Poliomyelitis	171	119	30.4% [23.9-36.6]
Pertussis	50	9	82.0% [69.5-90.5]

* % of people who thought that they were up to date and were not.

## Discussion

The VC rates obtained were similar to those observed in national and regional evaluations [6-8]. These authors analysed the methods and tools employed in France to ascertain vaccination coverage in adults and concluded that it was difficult to collect information on VC in adult populations through questionnaires. The best tool for assessing VC is serological testing, but it is very expensive and not easy to implement in population surveys.

The geographical and social diversity of participating laboratories ironed out any existing disparities. A recruitment bias could not be totally excluded, and recall bias was difficult to avoid. However, the likelihood that vaccination status was related to prescribed laboratory tests was very low and, in this case, selection bias would be limited. Care should be taken when generalising the results because the participating laboratories were not randomised.

The population interviewed was relatively elderly, retired and therefore more inclined to provide documentation by virtue of its greater availability. The population group likely to be parents (20–40 years) and reporting documentation (15.1%) was under-represented compared to that observed in Lyon city (47%). In addition, pertussis vaccination is not recommended during pregnancy in France, which does not allow us to compare the data to other countries.

Subjects agreeing to participate in the survey and to produce vaccination documentation might be more in tune with their health and more aware of the need to remain up to date with their vaccinations. A similar survey compared reported vaccinations with vaccinations confirmed by documentary evidence [9].

These findings might not be generalizable to the French population but may be representative of populations in large French cities (i.e. Paris, Marseille, and Strasbourg) or other large European cities.

dT-IPV VC decreases with age and requires the institution of vaccination campaigns among older people, underlining the importance of administering tetanus vaccine combined with other valences in accordance with official recommendations. Data collection tools for vaccination coverage appear to be incomplete and imprecise. Targeted surveillance to detect regional and social differences would be desirable [10].

## Conclusions

VC measurement should be a public health priority to improve health education campaigns. Our study underlined the need to highlight prevention messages relating to vaccination. Its main value was documentary evidence. Comparison of reported data with confirmed data revealed a considerable percentage of subjects who wrongly believed their vaccinations to be up to date, particularly for pertussis. The percentage of people who thought they were vaccinated but who were not should prompt health authorities to develop VC monitoring programmes to prevent insufficient vaccination due to ignorance of individual status. The collection of vaccination coverage data needs to be improved and could be centred in the community and/or hospital and/or workplace. Investigations should evaluate the characteristics of individuals who incorrectly report their vaccine status. This issue was not explored because of limited sample size. Improving vaccination coverage involves adapting measurement tools, developing public information campaigns, training doctors and ensuring a better understanding on the part of the general public.

## Competing interests

EC was an employee of Sanofi Pasteur MSD at study time. Sanofi Pasteur MSD provided financial support for study conduct. All other authors declare that they have no competing interests regarding this study.

## Authors’ contributions

DB, PV, CDS and EC contributed to study design DB and PV drafted the initial manuscript. JT provided access to laboratories. DB, EC and CDS contributed to data analysis and interpretation. All authors read, commented on and approved the final manuscript version.

## Pre-publication history

The pre-publication history for this paper can be accessed here:

http://www.biomedcentral.com/1471-2458/12/940/prepub
